# The Critical Appraisal of the Papers Published in the "Iranian Journal of Psychiatry and Behavioral Sciences", 2007-2010

**Published:** 2013

**Authors:** Mehrnoosh Kosaryan, Khadijeh Rabiei

**Affiliations:** 1 Professor, Department of Pediatrics AND Thalassemia Research Center, Mazandaran University of Medical Sciences, Sari, Iran.; 2 General Practitioner, Traditional and Complementary Medicine Research Center, Mazandaran University of Medical Sciences, Sari, Iran.

**Keywords:** Evidance Base Medicine, Critical Appraisal, Research Methodology

## Abstract

**Objective:** This study has been done in order to evaluate the papers published in the "Iranian Journal of Psychiatry and Behavioral Sciences" from 2007 to 2010.

**Methods:** A questionnaire was developed according to the design, evidence level, and recommendations to write scientific papers. Validity was achieved by consulting experts. Reliability was tested by re-evaluation of 7 randomly selected papers, one month after the first evaluation by Pearson correlation coefficient (r = 0.8). Different parts of the paper, including title, abstract, introduction, materials and methods, results, discussion, and references, were evaluated by a total of 47 questions. Each required item was judged as: appropriate, partially appropriate, not appropriate, and not applicable. SPSS software was used for descriptive analysis.

**Results: **From spring 2007 to summer 2010, 7 issues with 72 papers were published. The most frequent problem in the title was that one could not understand the design of the research by reading it. In the abstract part, in 90% of papers, time and setting of research were not mentioned. Statistical test was not mentioned in 70%, and reliability of the questionnaires was not mentioned in 70% of papers. The discussion part was the hardest part to judge and had few inappropriate issues, such as unnecessary repetition of introduction and/or results; in 20% of papers the conclusion was not appropriate based on the research design.

**Conclusions:** The evaluated papers had strong points, yet more effort is needed for them to approach excellence.

**Declaration of interest:** None.

## Introduction

In Iran and the Middle East, there is a tense competition for more publications in international journals (1). It is actually an important point for evaluation of research and scientific activities of universities and research centers (2). 

Moreover, career promotions are influenced by the number of publications rather than the evidence they have produced. Another important point is whether the journal is indexed in internationally validated sites, such as "Thomson Reuters (formerly ISI) Web of Science". Newer criteria for evaluation of publications such as impact factor of journals and citations to the papers are becoming more important as more and more journals join the validated indexes (3). 

Critical appraisal of published papers has many benefits. It works as a watch dog and decreases the pressure that is always present from authors to bypass stages for evaluation and correction, which prolong the time before publication. It should also be a routine effort to improve the quality of papers will be selected for future issues. It is more popular in western countries, but recently more of such papers are being published in Iranian journals (4-8). 

This article is the result of a study aimed to evaluate the papers published in the English journal of Mazandaran University of Medical Sciences "Iranian Journal of Psychiatry and Behavioral Sciences" dedicated to psychiatric and behavioral disorders under the Psychiatry and Behavioral Sciences Research Center, Sari, Iran.

## Materials and Methods

A descriptive study was done in summer 2010. All 7 published issues of the journal were evaluated according to research methodology, scientific writing, and evidence-based medicine references. The time and number of issues, number of editorial board members, different parts of the journal, and page numbers were collected. For each article, research design, number of authors, keywords, and references were reported. 

All original articles were evaluated after covering the name of the authors and coding (blinded evaluation). A questionnaire was developed. Its validity was achieved by consulting experts in research methodology and scientific writing. Reliability was tested by evaluation of 7 randomly selected papers, one month after the first evaluation. Same opinions were scored 1, and different opinions were scored 0. The sum of the scores of both evaluations was tested by Pearson correlation coefficient (r = 0.8). Different parts of the paper including title (1 item), abstract (7 items), introduction (4 items), materials and methods (18 items), results (6 items), discussion (6 items), references (3 items), and 2 general items regarding spelling and language skill were evaluated by a total of 47 questions. Items looked for in the abstract were whether the time and setting of research, research design, statistical tests, p-value for differences, clear answer for the main question, and an appropriate conclusion were mentioned.

Items for the introduction were as follows: clear explanation of the study including controversies and/or lack of knowledge, clear main objective, place of study, and logical sequence of statements. 

Items for the materials and methods part were: whether the research design, time of study, statistical tests, clear description of main outcome, and validity and reliability of measurements were mentioned. Whether appropriate tests were used, especially for ordinal variables which in basic are not parameters, and whether a pilot study was done. The explanation of number of studied cases, inclusive/exclusive criteria, randomization, and blindness of any kind if applicable were looked for. Duration of follow-up in cohort studies or longitudinal studies was another key point looked for. The side effects of intervention, patient satisfaction in clinical trials, and intention–to-treat in analysis of outcome were also included in the questionnaire. Any required item was judged as: appropriate, partially appropriate, not appropriate, and not applicable. Furthermore, the number of authors, keywords, and references were recorded. 

Items looked for in the results section, were: whether the main outcome was addressed clearly, all results presented in one form and in an appropriate way, and tables/figures were simple and correct, and any explanation or conclusion in the results section was judged as inappropriate. Specific statistics, if there were different kinds, need to be mentioned along with the p-value.

In the discussion section the point that was important to avoid was repetition of the introduction or result. Pointing out limitations of the study and disclosing new questions and future studies were judged as necessary. The discussion section needed to include the clinical validation of the results and if it had external validity. 

In the reference part use of Farsi papers was an appropriate point; however, references that were not used enough were considered inappropriate. The style of addressing the references was also paid attention to. SPSS for Windows (version 17, SPSS Inc., Chicago, IL., USA) was used for descriptive analysis.

## Results

It was a biannually published journal, its editorial board consisted of 24 distinguished professionals in the field, and in each issue about 20 others worked as referees. Each issue consisted of an editorial paper, a review article, 7 original researches, one case report, and a short report. Each issue had a few pages for selected abstracts from other journals and for events/announcements. Each issue had at least 50 to a maximum of 113 pages. 

Seventy two papers including 7 (9.7%) editorial, 45 (62.5%) based on original articles, 6 (8.3%) case reports, 5 (6.9%) brief reports, and 9 (12.5%) review articles were published. Original researches were descriptive in 39 (86.6%), clinical trials in 11 (24.4%), historical cohort in 4 (8.8%), and case–control in 2 (4.4%) of the cases. No papers were prospective cohort; there were no systematic reviews and all 9 review articles were expert opinions. The number of authors was 203 with the mean of 3.2 ± 1.7, and keywords and references were 3.4 ± 1.5 and 25.1 ± 3, respectively. 

Three papers which were introduced as "qualitative" were excluded from the study, because the questionnaire was invalid for this design. 

The most frequent problem in the title was that one could not understand the design of the research by reading it. In the abstract part, in 90% of papers time, and in 60% of them the setting of the research was not mentioned. 

Statistical test was not mentioned in 67%, and reliability of the questionnaires was not mentioned in 64% of papers. In 20%, tests which were appropriate for continuous variables were used for ordinal variables. 

The result part had the least problems. The discussion part was the hardest part to judge and had few inappropriate issues, such as unnecessary repetition of introduction and/or results. In 22%, the conclusion was not appropriate based on the research design. 

In 50% of papers, at least one reference to an Iranian research was present. All references were ordered based on the Vancouver system and rare typing mistakes were noted. 

Results of different parts are shown in tables 1-6. The spelling skill and language skill in 71.5%, and 28.5% of papers were appropriate, respectively.

**Table 1:** Original papers appraisal of the abstract part according to items looked for

**Items**	**Appropriate** **n (%)**	**Partially appropriate** **n (%)**	**Not appropriate** **n (%)**	**Not applicable** **n (%)**
**Time of research**	04 (10.3)	0 (0)0	35 (89.7)	0 (0)
**Place of research**	15 (38.5)	0 (0)0	24 (61.5)	0 (0)
**Name of method**	19 (48.7)	03 (7.7)	17 (43.6)	0 (0)
**Statistical test**	05 (12.8)	08 (20.5)	26 (66.7)	0 (0)
**P-value**	12 (30.8)	0 (0)0	25 (64.1)	2 (5.1)
**Appropriate conclusion**	13 (36.1)	15 (41.7)	08 (22.2)	0 (0)
**Clear answer**	07 (17.9)	27 (69.2)	05 (12.8)	0 (0)

**Table 2.** Original papers appraisal of the introduction part according to items looked for

**Items**	**Appropriate** **n (%)**	**Partially appropriate** **n (%)**	**Not appropriate** **n (%)**
**Clear explanation**	10 (25.6)	18 (46.2)	11 (28.2)
**Clear main objectives**	14 (35.9)	11 (28.2)	14 (35.9)
**Place of study**	14 (35.9)	0 (0)0	25 (64.1)
**Logical sequence of statement**	0 (0)0	8 (20.5)	31 (79.5)

**Table 3:** Original papers appraisal of the "Materials and Methods part according to items looked for

**Items**	**Appropriate** **n (%)**	**Partially appropriate** **n (%)**	**Not appropriate** **n (%)**	**Not applicable** **n (%)**
**Name of research design**	17 (41.5)	01 (2.4)	23 (56.1)	0 (0)0
**Time of study**	15 (35.7)	0 (0)0	27 (64.3)	0 (0)0
**Statistical test**	11 (27.5)	24 (60.0)	05 (12.5)	0 (0)0
**Main outcome**	05 (12.5)	17 (42.5)	18 (45.0)	0 (0)0
**Explanation of number of study**	13 (31.7)	13 (31.7)	15 (36.6)	0 (0)0
**Appropriate test for ordinal variables**	08 (20.0)	0 (0)0	32 (80.0)	0 (0)0
**All Statistical tests**	1 (2.7)	10 (27.0)	26 (70.3)	0 (0)0
**Validity**	13 (31.0)	02 (4.8)	27 (64.3)	0 (0)0
**Reliability**	12 (28.6)	03 (7.1)	27 (64.3)	0 (0)0
**Explanation of number of studied cases**	0 (0)0	02 (4.8)	40 (95.2)	0 (0)0
**Inclusive criteria**	07 (17.1)	16 (39.0)	18 (43.9)	0 (0)0
**Exclusive criteria**	05 (11.9)	08 (19.0)	29 (69.0)	0 (0)0
**Randomization**	1 (2.4)	4 (9.5)	3 (7.1)	34 (81.0)
**Blindness**	1 (2.4)	2 (4.8)	05 (11.9)	34 (81.0)
**Duration of follow-up**	1 (2.4)	05 (12.2)	1 (2.4)	34 (82.9)
**Side effect**	1 (2.4)	2 (4.8)	4 (9.5)	35 (83.3)
**Patient satisfaction**	0 (0)0	0 (0)0	07 (16.7)	35 (83.3)
**Intention to treat**	1 (2.4)	0 (0)0	07 (16.7)	34 (81.0)

**Table 4:** Original papers appraisal of the results part according to items looked for

**Items**	**Appropriate** **n (%)**	**Partially appropriate** **n (%)**	**Not appropriate** **n (%)**	**Not applicable** **n (%)**
**Consider the main result**	16 (40.0)	15 (37.5)	9 (22.5)	0 (0)
**One result, appropriate way**	35 (87.5)	0 (0)	5 (12.5)	0 (0)
**Correct tables**	19 (48.7)	5 (12.8)	12 (30.8)	3 (7.7)
**Correct figures**	4 (10.3)	3 (7.7)	1 (2.6)	31 (79.5)
**Presence of conclusion in result section**	30 (76.9)	3 (7.7)	6 (15.4)	0 (0)
**Specific statistics, P-value**	12 (30.0)	10 (25)	16 (40)	2 (5)

**Table 5:** Original papers appraisal of discussion part according to items looked for

**Items**	**Appropriate** **n (%)**	**Partially appropriate** **n (%)**	**Not appropriate** **n (%)**
**Repetition of the introduction**	23 (62.2)	0 (0)	14 (37.8)
**Repetition of the result**	21 (55.3)	0 (0)	17 (44.7)
**Limitation of study**	21 (53.8)	0 (0)	18 (46.2)
**Disclose new questions**	10 (26.3)	0 (0)	28 (73.7)
**Clinical relevancy**	8 (21.1)	21 (55.3)	9 (23.7)
**External validity**	2 (5.7)	2 (5.7)	31 (88.6)

**Table 6:** Original papers appraisal of reference part according to items looked for

**Items**	**Appropriate** **n (%)**	**Partially appropriate** **n (%)**	**Not appropriate** **n (%)**
**Farsi paper**	20 (51.3)	0 (0)	19 (48.7)
**Inappropriate reference**	27 (71.1)	3 (7.9)	8 (21.1)

## Discussion

The current study showed that in a large number of articles, the title was not representative of design, and in other words, the main question of the study. Cross-sectional studies are especially dependent on place and time of research. The part which is being read the most is the abstract section. As there usually exists a word limit for the abstract, one must use the least words to represent the work that has been done, which is why it should be accurately structured in most journals. For many papers this part is the only section which is available for everyone free of charge. In addition, in seminar summaries the abstract is the only published text. If the authors do not use this opportunity, especially in materials and methods, other colleagues will not be interested in the full text form. As a result, citation to the paper will not happen. One of the most important parts of an abstract is the conclusion that should be in accordance with the goal or the main question of the study. Obviously, based on a cross-sectional study one cannot recommend a treatment or preventive intervention. Sometimes, authors use their previous knowledge to conclude the study. The cornerstone of introduction is to justify the study. On the other hand, if all references were discussed completely nothing would remain for the discussion. One of the most important points in writing the introduction is that one should address the problem directly. One of the most frequent mistakes is that the introduction begins with the risk factor not the problem. Some authors start with some dramatic statements which are not related to the problem; this is usually boring and induces a negative attitude in readers. The most critical part of a paper which acquires the confidence of professionals and convinces them to refer to it is the material and method part. There should be no limits in the word count, because, especially in clinical trials, there are many points to be addressed in detail. However, if it is possible by referencing to previous works, one can save the space; on the condition that, the reference is readily available in terms of language and distribution. Starting with the research design type is useful in order to increase the reader's anticipation. Instruments that were used for research have crucial importance. In behavioral studies, the questionnaires are the main instrument. If they are produced with a different language and in a different culture, they should become valid and reliable for the specific population (9-13). 

One delicate aspect of psychological studies is that making scores for specific definitions do not allow us to use parametric statistical tests for a variable with ordinal scale (14). Parametric tests are not allowed in all continuous variables, if normal distribution of data is not achieved and/or random sampling was not practiced (15). Sampling is random in none of the analytical or interventional studies. In contrast, sampling is according to the goal of the research. 

Using Student's t-test instead of Mann-Whitney u test, especially if the number of cases is more than 25, can show significant differences, which is not clinically significant. The number of studied cases directly influences the external validity of the result. Explanation for the number depends on the type of design, heterogeneity of the study population, and practical definitions, and the power, the least expected odds ratio, and the accepted errors. Regarding the presentation of the results, one has to choose the form that is more informative and takes up less space. Any explanation of data or mentioning methods for measurements should be avoided in this part.

The discussion part is the part that shows the ability of authors to explain the data, and address the validity and clinical importance of results. Repeating the introduction and/or result is considered as lack of the ability to use existing literature and logic to address the conclusion. Professionals could judge this part well. Peer-reviewed journals benefit referees' opinion in this regard. Not all specialists are experts in research methodology; so, it is recommended that the editorial board have members who are experts and experienced in research methodology. Unfortunately, statisticians do not necessarily possess this quality.

In order to simplify management of the references, one may use the Harvard system initially and then change it to the Vancouver system, which is more popular, by one of the reference manager software. A question that should be addressed is that "to what extend does the editor have the right to ask for changes?" Raising scientific standards of the journal, which is the responsibility of the editorial board, and chief editor in particular, puts an end to too many back and forth communications and prolongation of the acceptance process which results in authors' dissatisfaction. On the other hand, publishing papers soon but with obvious mistakes pleases the authors but makes the journal vulnerable in regard to international validation (16-20).
